# A Case of Gastrointestinal Stromal Tumour (GIST) in the Duodenum in a Young Adult

**DOI:** 10.7759/cureus.53331

**Published:** 2024-01-31

**Authors:** Dinesh Abhijeeth Shanker, Sampath Kumar, Ahmed Al-Mukhtar, Asha Dube, Nehemiah Samuel

**Affiliations:** 1 General Internal Medicine, Doncaster Royal Infirmary, Doncaster, GBR; 2 Gastroenterology and Hepatology, Doncaster Royal Infirmary, Doncaster, GBR; 3 Gastrointestinal Surgery, Sheffield Teaching Hospitals National Health Service (NHS) Foundation Trust, Sheffield, GBR; 4 Histopathology, Sheffield Teaching Hospitals National Health Service (NHS) Foundation Trust, Sheffield, GBR; 5 Gastrointestinal Surgery, Doncaster Royal Infirmary, Doncaster, GBR

**Keywords:** gastrointestinal bleed, gastrointestinal neoplasms, duodenal cancer, oesophagogastroduodenoscopy (ogd), gastrointestinal stromal tumor (gist)

## Abstract

Gastrointestinal stromal tumours (GISTs) are rare gastrointestinal (GI) malignancies, but the most prevalent mesenchymal tumours of the GI tract arise from the interstitial cells of Cajal. They account for 1-3% of all GI malignancies, and only 3-5% of all cases of GIST are located at the duodenal. We present a case of a young adult who presented to the ED with symptoms of GI bleeding.

## Introduction

Gastrointestinal stromal tumours (GISTs) are gastrointestinal (GI) tumours originating from the interstitial cells of Cajal (ICC). While they account for only 1-3% of all GI cancers, they are the most prevalent mesenchymal tumour found in the GI system [1]. In the United Kingdom. Annual incidence is estimated to be between 1.32 and 1.50 per 100,000 people, according to Starczewska et al., while globally, the incidence is between 10 and 15 per million people, as per the systematic review conducted by Soreide et al. [2-3]. The risk of developing GIST increases with age, and the highest occurrence is noted in the age group between 70 and 79 years [1]. Under 10% of all GISTs are diagnosed in under 40s [4].

GISTs are most frequently found in the stomach, accounting for 60-70%, followed by the small intestine, which contributes roughly 20-30% of cases. The remaining cases are distributed across various locations within the GI tract, including the oesophagus, colon, rectum, and extraintestinal sites such as the pancreas and omentum [1,4]. The clinical presentation will depend on the anatomic location of the GIST along with the size of the lesion. Patients usually present with symptoms when the size of the GIST is >5 cm or has impinged on another structure. Common symptoms include abdominal pain and distension, palpable mass, early satiety, nausea and vomiting, and symptoms of anaemia. 40% of the patients may present with upper GI bleeds or intraperitoneal bleeds [1,5]. The tumours can metastasize, with the liver being the most common, followed by the peritoneal cavity. Spread to lymph, lungs, and bone nodes is relatively rare [1,6]. Less than 5% of GISTs can be familial and be associated with other syndromes such as neurofibromatosis type 1, Carney's triad, or the Carney-Stratakis triad [7].

We would like to present a case of GIST, a rare GI malignancy with an incidence of less than 10% in individuals under the age of 40 [4]. Furthermore, duodenal GISTs are even rarer, accounting for only 3-5% of all GIST cases [8]. This unique combination of factors is what makes our case particularly intriguing and uncommon.

## Case presentation

A 30-year-old male, otherwise fit and well with a background history of asthma, initially presented to the ED with a one-day history of bloody diarrhoea and vomiting. Initially, he started vomiting, which was watery; however, he later had a few streaks of blood. This was followed by diarrhoea, which was mixed with blood. His symptoms developed a day after consuming a sandwich from a takeaway restaurant. Symptoms appeared to have settled with antiemetic and intravenous fluids, and he was discharged from the ED with a diagnosis of infective gastroenteritis and a possible Mallory-Weiss tear. However, on his way back home, he had a further episode of dark and bloody vomiting. Throughout the night, he continued to have bloody diarrhoea and vomiting, prompting him to return to the hospital. At the time of the second presentation, he was referred to the surgeons as a potential lower GI bleed. Blood investigations during this admission are mentioned in Table 1

**Table 1:** Relevant blood investigations

	2017 (previous baseline)	Day 1: 1st presentation to the emergency department (ED)	Day 2: 2nd presentation to the emergency department (ED)	Day 3	Day 4
Haemoglobin (126-180) g/l	164	125	98	84	69
Platelets (140-450) x 10^9^/L	-	239	236	151	117
White blood cells (4.0-12.0) x 10^9^/L	-	-	-	-	-
Creatinine (64-104) umol/L	68	79	70	-	68
Urea (2.5-7.8) mmol/L	4.9	10.5	11.7	-	4.5

**Figure 1:**
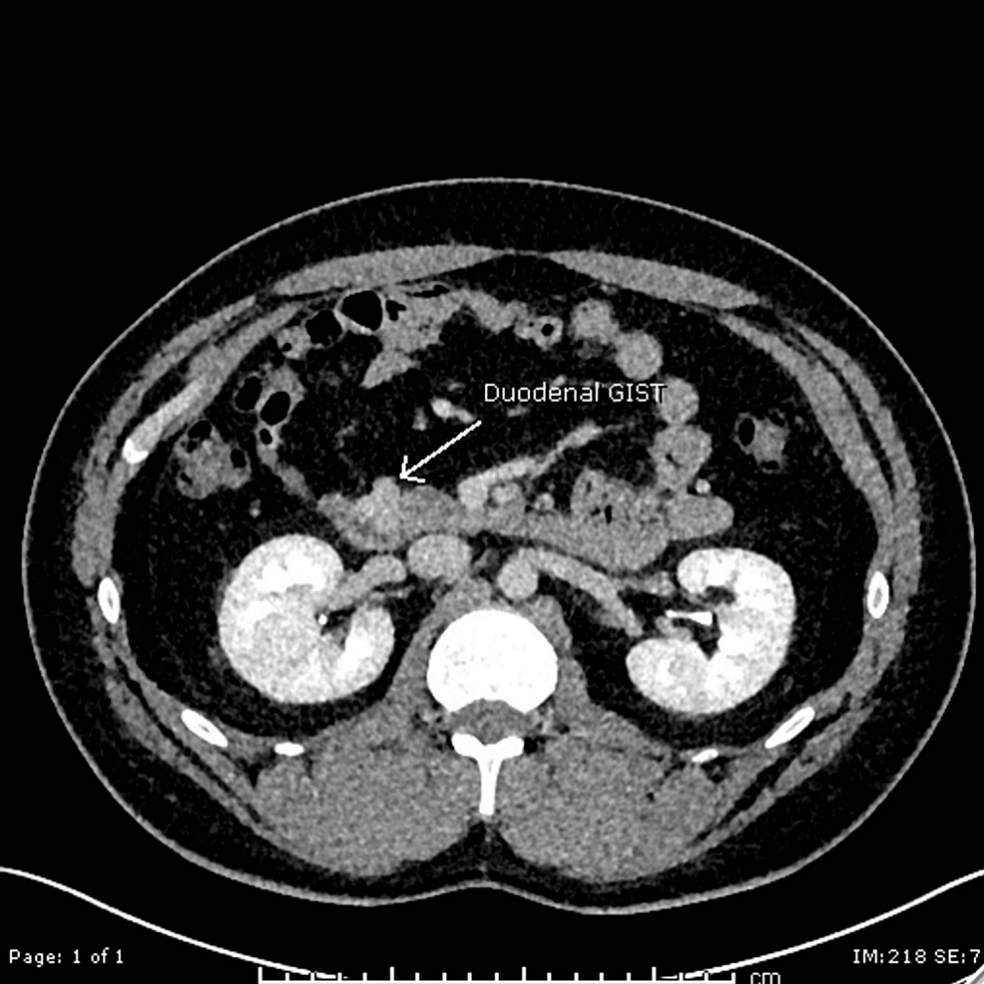
Duodenal gastrointestinal stromal tumour (GIST) on computed tomography (CT) angiogram of the abdomen, coronal view

**Figure 2:**
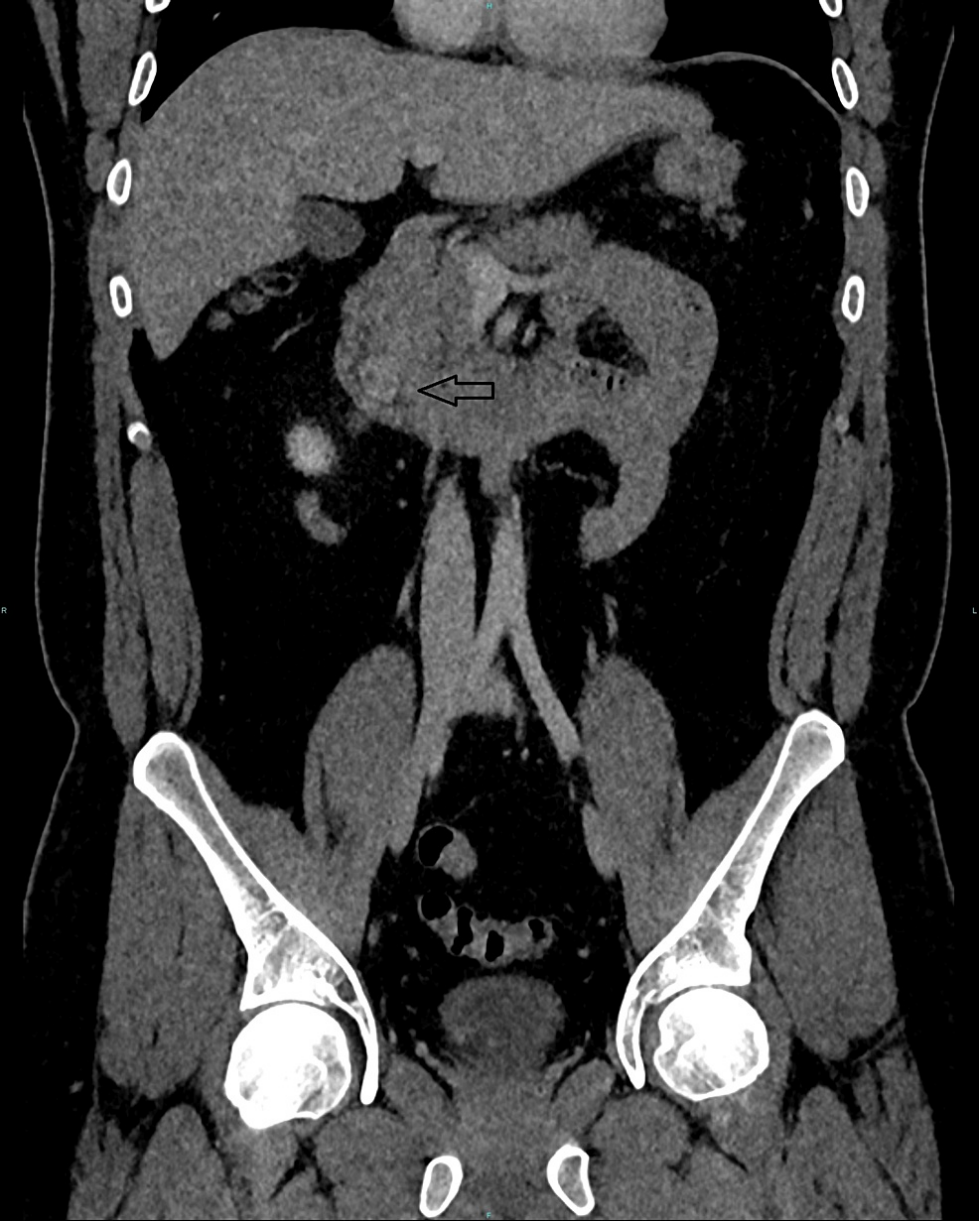
Duodenal gastrointestinal stromal tumour (GIST) on computed tomography (CT) angiogram of the abdomen, axial view Arrowhead points to the gastrointestinal stromal tumour (GIST)

Following surgical review, they proceeded with a CT scan angiogram (Figures 1-2) of the abdomen, which revealed a hypervascular intraluminal lesion related to the distal 2nd part of the duodenum with no convincing evidence of active bleeding. He was referred to the medical team for management as an upper GI bleed.

**Figure 3:**
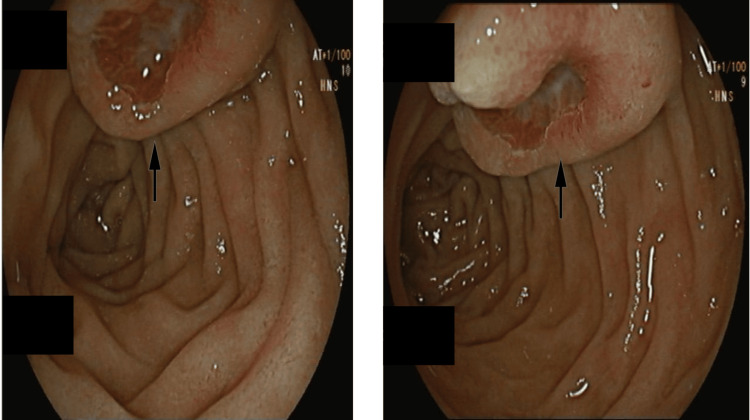
Lesion suggestive of gastrointestinal stromal tumour (GIST) on oesophagogastroduodenoscopy (OGD) marked by an arrow

**Figure 4:**
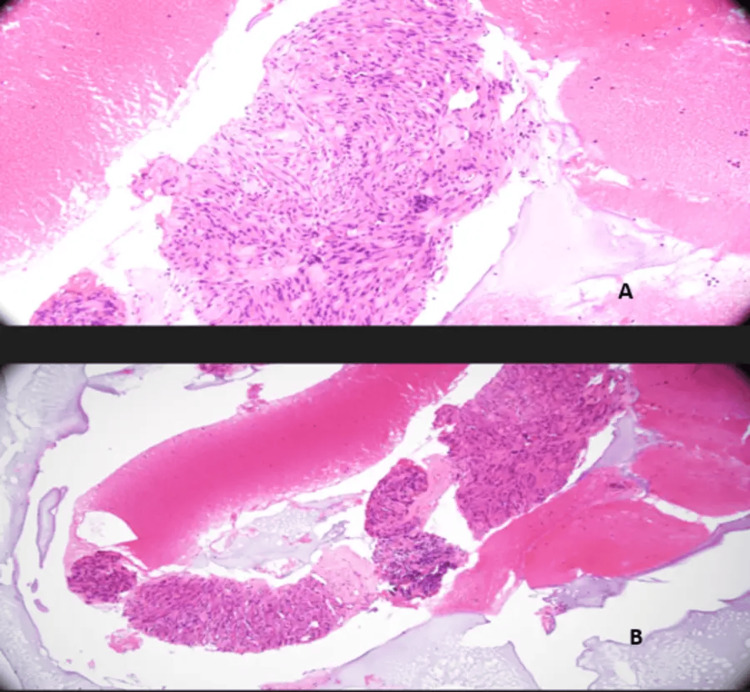
Fine needle biopsy of a submucosa from the lesion in the second part of the duodenum stained with haematoxylin and eosin A: Under low power microscope, the specimen contained cores of blood and slender cores of a spindle cell lesion. B: Under high power microscope, spindle cells are arranged in fascicles and nuclei are relatively uniform

**Figure 5:**
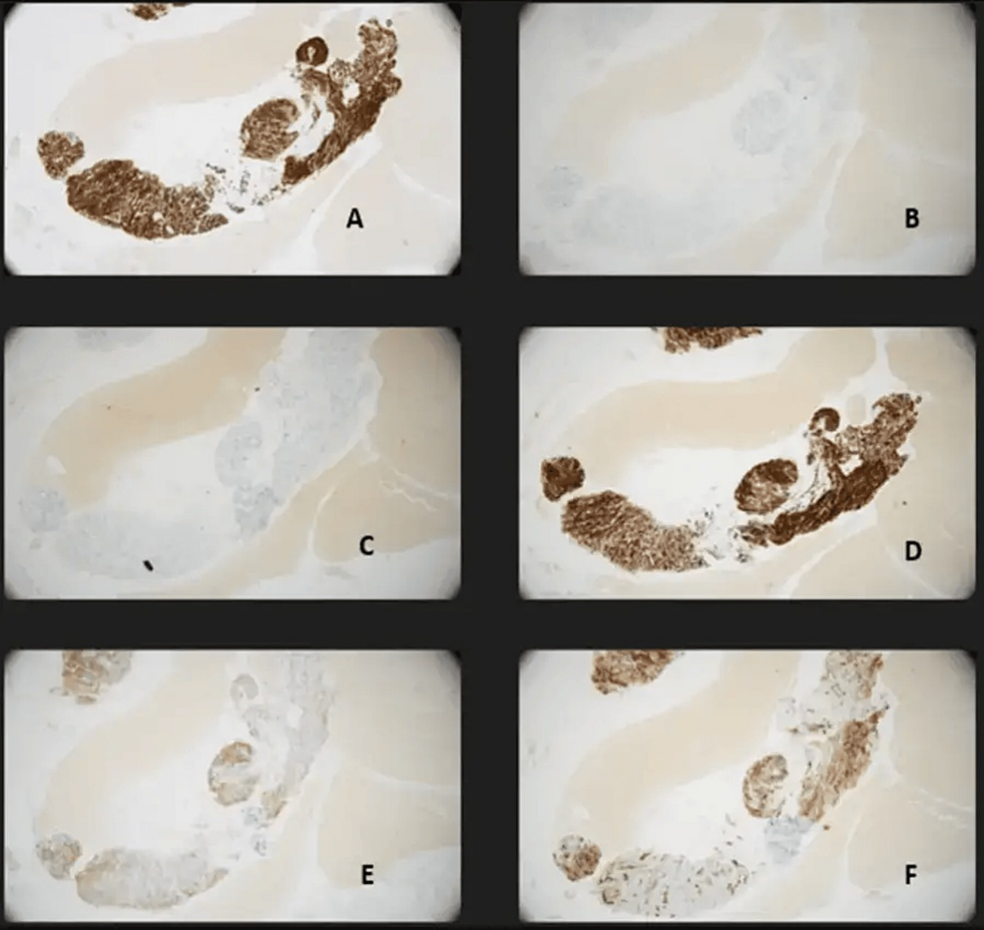
Immunohistochemistry of the lesion. Immunohistochemistry showed the lesion to be DOG1 positive, moderately CD117 (c-kit)+, and SMA+. D33 (confirming that this is not a smooth muscle tumour) and S-100 were negative (confirming that this is not of neural origin). DOG1 positivity confirms gastrointestinal stromal tumour (GIST). CD117 presence adds certainty and dictates possible treatment options. SMA may have seen some GISTs too A: DOG1 positive. B: S100 negative. C: D33 negative. D: SMA positive. E: CD117 positive. F: CD34 positive

He initially had an IV proton pump inhibitor, IV fluids, and 4 units of packed red cell transfusion as part of the management of the GI bleed. Subsequently, he underwent an urgent oesophagogastroduodenoscopy (OGD) within two hours of the CT scan. The first OGD revealed a 2-cm polypoidal lesion with central umbilication and ulceration, with fresh blood from the lesion in the 2nd part of the duodenum. It was suggestive of a GIST (Figure 3). To manage the likely source of haemorrhage, two haemostatic clips were applied, and three quadrants were injected with a total of 8 ml (1:10,000) of adrenaline. Additionally, haemospray was applied. He remained haemodynamically stable throughout and was treated with 72 hours of PPI infusion on the ward. A second OGD was performed five days after discharge to assess the lesion. The case was referred to the regional sarcoma multidisciplinary team (MDT). After evaluating the CT images, the MDT were of the opinion that the lesion had an intraluminal and extraluminal component to it. He underwent an endoscopic ultrasound for a biopsy. Biopsy results showed fragments of epithelial cells and the core of spindle cells (Figure 4). Immunohistochemical analysis of spindle cells showed that they stained CD117 (c-kit), DOG1, SMA, and CD34. The Ki-67 index was between 2 and 2.5 (Figure 5). All this was in keeping with GIST, without any malignant features. He is currently being worked up for a Whipple procedure.

## Discussion

The term GIST was first proposed by Mazur and Clark in the 1980s for all intra-abdominal tumours that were not cancerous but exhibited histology of smooth muscles and neural elements. However, in the late 1990s, with the advancement in molecular biology, it became apparent that these tumours closely resembled the pacemaker cells found in the GI tract, also known as interstitial cells of Cajal (ICC) [1].

Like other GISTs, the clinical presentation of duodenal GIST can vary. As the patient described in our case report, the most common presentation is a GI bleed. They exhibit a lower prevalence of expression of some of the markers of poor prognosis, such as p-16 loss, ki-67, and loss of p-53, when compared to other GISTs [9-10]. This suggests that they have a favourable outcome compared to other GISTs. Duodenal GISTs have a lower mitotic number and are generally smaller in size at the time of diagnosis, which may suggest that they have a more benign path compared to their cousins. However, all these studies were based on small cohorts of patients due to their lower incidence, and further confirmation through larger studies is needed [9].

It is noted that 85-95% of the GISTs have a mutation in the KIT gene and immunohistologic staining of CD117, while the remaining have a mutation in a similar tyrosine kinase named PDGFRA [11]. Exons 9 and 11 are commonly affected by KIT mutations, while mutations in exons 13, 17, and 18 are rarely observed. Similarly, exon 18 is most commonly identified in PDGFRA mutations. This information about the tumour is essential for both prognostic and therapeutic benefits [11]. Duodenal GISTs do not immunohistochemically differ from other GISTs [9].

The management of duodenal GIST will depend on the size of the tumour and the extent of infiltration. Local resection is considered if the tumour size is less than 2 cm and the ampulla of Vater and common bile duct can be preserved. If this cannot be achieved, a pancreaticoduodenectomy is performed. Performing a local resection may be associated with a better quality of life; however, there is a chance that the tumour can recur if adequate resection is not achieved. Although pancreaticoduodenectomy is a more challenging surgery, needing greater skill and technical expertise, it is preferred in cases with a larger tumour size and a higher chance of recurrence. Imatinib mesylate is a tyrosine kinase inhibitor [9]. It can be used as a neoadjuvant, an adjuvant chemotherapeutic drug, or for local recurrence.

## Conclusions

Through this case, we wish to highlight this rare combination, i.e., a young adult presenting with a rare GI malignancy in an uncommon location, and highlight this as a possibility. Clinical features of GIST can vary depending on the location and size. The treatment depends on the size and the anatomical relationship of the tumour and its structures. With the advancement in molecular biology and genetics, we now have chemotherapeutic agents that may be utilised.
